# Development of a quality assessment tool for systematic reviews of observational studies (QATSO) of HIV prevalence in men having sex with men and associated risk behaviours

**DOI:** 10.1186/1742-7622-5-23

**Published:** 2008-11-17

**Authors:** William CW Wong, Catherine SK Cheung, Graham J Hart

**Affiliations:** 1 Department of General Practice, 200 Berkeley Street, Carlton, Vic 3053, Australia; 2 Department of Community and Family Medicine, the Chinese University of Hong Kong, Shatin, New Territories, Hong Kong, PR China; 3 Centre for Sexual Health and HIV Research, University College London, Mortimer Market Centre (off Capper St), London, WC1E 6JB, UK

## Abstract

**Background:**

Systematic reviews based on the critical appraisal of observational and analytic studies on HIV prevalence and risk factors for HIV transmission among men having sex with men are very useful for health care decisions and planning. Such appraisal is particularly difficult, however, as the quality assessment tools available for use with observational and analytic studies are poorly established.

**Methods:**

We reviewed the existing quality assessment tools for systematic reviews of observational studies and developed a concise quality assessment checklist to help standardise decisions regarding the quality of studies, with careful consideration of issues such as external and internal validity.

**Results:**

A pilot version of the checklist was developed based on epidemiological principles, reviews of study designs, and existing checklists for the assessment of observational studies. The Quality Assessment Tool for Systematic Reviews of Observational Studies (QATSO) Score consists of five items: External validity (1 item), reporting (2 items), bias (1 item) and confounding factors (1 item). Expert opinions were sought and it was tested on manuscripts that fulfil the inclusion criteria of a systematic review. Like all assessment scales, QATSO may oversimplify and generalise information yet it is inclusive, simple and practical to use, and allows comparability between papers.

**Conclusion:**

A specific tool that allows researchers to appraise and guide study quality of observational studies is developed and can be modified for similar studies in the future.

## Introduction

Epidemiological evidence-based research is becoming an increasingly important basis for health care decisions and planning. There is a dearth of reviews of observational and analytic studies on HIV prevalence and risk factors for HIV transmission among men having sex with men (MSM), and this is particularly the case in mainland China [1-3]. We sought to conduct a rigorous systematic review summarising HIV prevalence data in MSM and to measure their associated high risk behaviours in China, with the aim of providing systematic and comprehensive data for policymakers to devise appropriate plans for health promotion and interventions to control the spread of HIV in the target population.

A number of consensus statements have previously been prepared to encourage higher quality of reporting, including recommendations for reporting systematic reviews (QUOROM)[4], randomized trials (CONSORT)[5], studies of diagnostic tests (STARD)[6], meta-analyses of observational studies (MOOSE)[7] and observational epidemiological studies (STROBE)[8,9]. However all these were aimed at authors of reports, not at those seeking to assess the validity of what they read [10]. Of particular relevance here are the MOOSE and STROBE statements, both of which were developed as checklists designed to assist authors when writing up analytical observational studies, to support editors and reviewers when considering such articles for publication, and to help readers when critically appraising published articles [8]. However, there remains a clear disparity between the quality of tools available to aid the critical appraisal of observational studies when compared with those available for controlled trials, making the systematic review of the former particularly difficult. We believe a quality assessment tool is the key to any systematic review as it allows original research to be objectively appraised and evaluated, in order to inform subsequent decisions regarding inclusion by evaluating, ranking, or scoring the relevant studies [11-13].

A study conducted by Mallen and his coworkers [14] in 2006 revealed that quality assessment tools were grossly under-utilised in the evaluation of observational studies, in that only 13 out of 40 articles in 2003–2004 using published checklists/quality assessment tools such as NHS CRD [15,16], MOOSE [7], Downs and Black checklist [17] and Ottawa-Newcastle tool [18]. Of such tools, the Newcastle-Ottawa Scale (NOS) is one of the more comprehensive instruments for assessing the quality of non-randomised studies in meta-analyses: the 8-item instrument consists of three subscales, namely, selection of subjects (4-item), comparability of subjects (1-item), assessment of outcome/exposure (3-item). Despite having been recommended by the Cochrane Non-Randomized Studies Methods Working Group [19], it is only partly validated and primarily used to appraise cohort studies and case-control studies [18]. In short, our major challenge is that each study is to some extent unique, and that a quality checklist may consequently not include items that may be considered relevant for the purposes of the intended meta-analysis. We therefore set out to develop a concise quality assessment checklist to help standardise decisions regarding the quality of studies, with careful consideration of issues such as external and internal validity.

## Results and discussion

### Algorithm

Often both internal and external validity are assessed together during methodological quality assessment as interpretation of the findings of a study depends on design, conduct and analyses (internal validity), as well as on populations, interventions and outcome measures (external validity). The information gained from quality assessment is crucial in determining the strength of inferences and in assigning grades to recommendations generated within a review.

Our team proposed to identify case-control studies, cross-sectional studies with case-control design in the questions, and those intervention studies that address prevalence rates. A pilot version of the checklist was developed based on epidemiological principles, reviews of study designs, and existing checklists for the assessment of observational studies. It was later modified in light of preliminary and pilot application. The final tool, abbreviated as QATSO Score, covers the following aspects (Additional file 1):

1) External validity (1 item) – addresses the extent to which the findings from the study can be generalised to the population from which the study subjects are derived.

2) Reporting (2 items) – assesses whether the information provided in the paper is sufficient to allow a reader to make an unbiased assessment of the findings of the study. One of the items is specific for prevalence studies.

3) Bias (1 item) – addresses bias in the measurement of the outcomes in a study.

4) Confounding (1 item) – addresses whether studies have applied adjustment for confounding in the analysis. This item is specific to studies concerning association of risk factors.

Although the QATSO Score consists of five items, users may select 4–5 items depending on the type of studies being evaluated. Studies achieving 67% or more in the score will be regarded as "good" quality; 34–66% "fair"; and, below 33% "poor".

## Testing

Experts from the Hong Kong Branch of the Chinese Cochrane Centre and local HIV researchers (see acknowledgement) were invited to provide comments on the content validity of the assessment tool. This assessment tool was then pilot-tested with two independent reviewers to test the consistency of study quality. The two reviewers were asked to assess 10 observational studies selected at random from a group of 30 identified during a systematic review of HIV prevalence in MSM and associated risk factors. The reviewers were given guidance with regard to the interpretation of the items included in the checklist before reviewing the papers. Inter-rater reliability was shown to be good (Pearson coefficient = 0.86).

In order to evaluate the practicality of the tool, the time used to assess each paper was recorded. Both reviewers reported that they took an average of 10.4 ± 4.6 minutes to assess one paper with QATSO Score as compared to 23.0 ± 4.5 (p < 0.001) spent applying a validated lengthy checklist (comprising of 27-items) reported elsewhere [11].

## Implementation

We searched articles published in English and Chinese languages in the following electronic databases: MEDLINE (1966 to December 2006), EMBASE (1980 to December 2006), ProQuest Social Science Journal (1989 to December 2006), Anthropology (1984 to 1996), China Journal Net (1994 to December 2006) and Wan Fang Data (1998 to December 2006). To retrieve publications reporting HIV prevalence and risk behaviours among MSM in Mainland China, we performed a combined search strategy that included the following terms as both medical subject heading (MeSH) terms and text words: "prevalence", "epidemiology", "HIV infections", "Acquired Immunodeficiency Syndrome", "AIDS", "MSM", "male having sex with male", "men having sex with men", "men who have sex with men", "homosexuality, male", "gay", "homosexual", "bisexual", "queer", "male sex worker", "male sexual worker", sexual risk behaviour", sexual behaviour", "risk taking", "risk factors", "protective factors", "China" and "Tibet". We manually searched for review articles and abstracts from the reference list of identified articles. Additional reports from known experts in field through our contacts and professionals were included for review.

Data were independently abstracted onto a standardized form by two independent reviewers. Data abstracted included study design, time period of study, place of origin, study setting, HIV prevalence, information source for exposure measurement, total number of persons in each group, odds ratio or risk ratios, with and without adjustment for potential. Conflicts in data abstraction were resolved by consensus. Data reporting conforms to the Meta-analysis of Observational Studies in Epidemiology (MOOSE) study group guidelines [7]. QATSO is then applied to assess the standard of each paper that fulfils the inclusion criteria.

During this process, we found that QATSO may over-simplify and generalise information one could extract from a published manuscript, an issue inherent in all quality assessment tools. For example, the relative importance of individual items will be lost through a summation of items represented by a total score. A careful balance has to be struck so that the final scale is inclusive and allows comparability between papers, yet is simple and practical to use. Secondly, any attempts at summarising quality on, for example, the inclusion or exclusion of a particular item, will invariably lose the significance of that item's magnitude. For example, a reported response rate *per se *does not necessarily mean that the response rate is satisfactory (item three in the scale); we therefore selected an arbitrary 60% response rate as a cut-off for acceptable quality. However, it is important to emphasise that the objective of this tool is to appraise and guide study quality; actual analyses are conducted in the next phase of systematic review or meta-analysis which will be reported elsewhere.

## Conclusion

Few quality assessment tools for the systematic review of observational studies are available and relevant for HIV prevalence in MSM and associated risk behaviours. We have developed a specific tool that researchers who wish to conduct similar systematic reviews can adopt to ensure that studies reach a level of quality that permit their inclusion on meta-analyses.

## Competing interests

The authors declare that they have no competing interests.

## Authors' contributions

WCWW conceived the original idea for the study, was involved in the design and testing of the QAT, and in the drafting and write-up of the final report. CSKC was involved in the testing of the QAT, as well as conducting the literature search and contributing to the write-up of the final report. GH was involved in designing the QAT, as well as in the preparation and writing of the final report. All authors have read and approved the manuscript.

## Supplementary Material

Additional file 1 **Quality assessment checklist for observational studies (QATSO Score) concerning HIV prevalence/risk behaviours among MSM.** Click here for file
