# Supplementary material for: Development of a quality assessment tool for systematic reviews of observational studies (QATSO) of HIV prevalence in men having sex with men and associated risk behaviours
Source: Emerg Themes Epidemiol. 2008 Nov 17;5:23. doi: 10.1186/1742-7622-5-23 (PMC2603000; doi:10.1186/1742-7622-5-23)
Supplement: Additional file 1 — Quality assessment checklist for observational studies (QATSO Score) concerning HIV prevalence/risk behaviours among MSM. [file 1742-7622-5-23-S1.doc]

**Additional File 1. Quality assessment checklist for observational studies (QATSO Score) concerning HIV prevalence/ risk behaviours among MSM**

1. Was the sampling method representative of the population intended to the study?

| A. | Non-probability sampling (including: purposive, quota , convenience and snowball sampling) | 0 |
| --- | --- | --- |
| B. | Probability sampling (including: simple random, systematic, stratified g, cluster, two-stage and multi-stage sampling) | 1 |

1. Was the measurement of HIV objective (if the article is focusing only on risk behaviour among MSM, please select “Not applicable” for this question)?

| A. | By questionnaires (Self-reported) | 0 |
| --- | --- | --- |
| B. | By clinical records or lab tests | 1 |
| C. | Not applicable | NA |

1. Did the study report any response rate? (If the reported response rate is below 60%, the question should be answered “No”.)

| A. | No | 0 |
| --- | --- | --- |
| B. | Yes | 1 |

1. Did the investigator(s) control for confounding factors (e.g. stratification/ matching/ restriction/ adjustment) when analyzing the associations (if the study contains purely descriptive results, no association and prediction tests were conducted in the test, please select “Not applicable”)?

| A. | No | 0 |
| --- | --- | --- |
| B. | Yes | 1 |
| C. | Not applicable | NA |

1. Was privacy or sensitivity of the nature of HIV considered when the survey was conducted eg if conducted in a non-MSM or general clinic setting?

| A. | No | 0 |
| --- | --- | --- |
| B. | Yes | 1 |

Scoring method: Total score divided by total number of all applicable items

Grading of the QACO score:

| 0% -33% | 33%- 66% | 67% -100% |
| --- | --- | --- |
| Bad | Satisfactory | Good |
